# Participation and consultation engagement strategies have complementary roles: A case study of patient and public involvement in clinical practice guideline development

**DOI:** 10.1111/hex.13018

**Published:** 2019-12-29

**Authors:** Melissa J. Armstrong, Gary S. Gronseth, Anna R. Gagliardi, C. Daniel Mullins

**Affiliations:** ^1^ Department of Neurology University of Florida College of Medicine Gainesville FL USA; ^2^ Department of Neurology University of Kansas Medical Center Kansas City KS USA; ^3^ Toronto General Research Institute University Health Network Toronto ON Canada; ^4^ Pharmaceutical Health Research Department University of Maryland School of Pharmacy Baltimore MD USA

**Keywords:** amyloid PET imaging, clinical practice guidelines, dementia, guideline adherence, guidelines as topic, patient and public involvement, patient participation, patient‐centred care

## Abstract

**Background:**

Patient and public involvement (PPI) is recommended when developing high‐quality clinical practice guidelines, but the effects of different PPI strategies are largely unstudied.

**Objective:**

To assess the impact of participation and consultation strategies on guideline question development.

**Design:**

Instrumental case study design.

**Setting and participants:**

This study used a clinical practice guideline in development by the American Academy of Neurology. A patient, two caregivers and a dementia advocate participated in the guideline development group alongside clinicians. The guideline protocol was posted for public consultation for 30 days.

**Interventions studied:**

Participation (patient representatives on the guideline development group) and consultation (public comment, survey) PPI strategies.

**Main outcome measures:**

Public comment responses and guideline development group meeting transcripts were analysed descriptively. Transcript quotes were compared to the conceptual model of PPI in guideline development. The effects of participation and consultation strategies within the guideline case were compared.

**Results:**

Participation strategies shaped discussions, set a patient‐centred scope, highlighted personal aspects of disease, affected how professionals viewed PPI, identified issues overlooked by medical professionals, and contributed to selecting patient‐relevant guideline populations and outcomes. Professionals responded to public comment more than patient representatives. Patient survey participants confirmed the priorities voiced by patient representatives on the guideline development group. Final guideline questions included populations and outcomes promoted by patient representatives despite negative feedback from professional public commenters.

**Discussion and conclusions:**

Participation and consultation PPI strategies have different advantages. Congruence between strategies increases the strength of the patient voice. Guideline developers should prioritize using both strategies for successful PPI.

## 1 INTRODUCTION

Patient and public involvement (PPI) is when patients, patient representatives (eg caregivers, advocates) and/or members of the public are actively involved in the development, conduct and implementation of activities in health care, such as research and health‐care policy. PPI is internationally recognized as an important contributor to quality development of clinical practice guidelines (hereafter called ‘guidelines’). Numerous organizations recommend or require that guideline developers engage health consumers, patients and/or patient representatives in guideline development, including the Guidelines International Network,^1^ the United States’ Institute of Medicine (IOM, now renamed the National Academy of Medicine)^2^ and the United Kingdom's National Institute for Health and Care Excellence.^3^ Both the Appraisal of Guidelines for Research and Evaluation II instrument^4^ and the Guideline Trustworthiness, Relevance and Utility Scoring Tool^5^ assess diversity in stakeholder involvement, including the presence of patient representatives. PPI in guidelines is advocated because it leads to the development of more patient‐centred and trustworthy guidelines, recognizes patients as experts, respects citizen rights in developing health policy, and empowers and informs consumers making health‐care decisions.^6^

**Figure 1:**
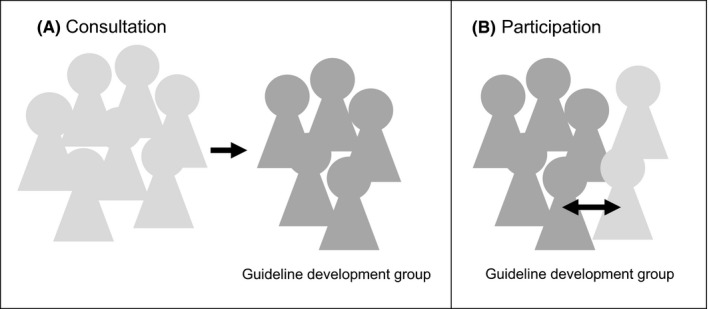
Consultation vs participation engagement strategies. Light grey: Patient and public stakeholders, dark grey: professional stakeholders. A, In consultation strategies, information flows from patient and public stakeholders to the guideline development group. Often consultation strategies engage a large, representative population. B, In participation strategies, patient and public stakeholders are active and equal participants in the guideline development group with information shared in both directions. Participation often involves only a small number of stakeholders

Consultation and participation are the two primary mechanisms for PPI in guidelines.^7^ These strategies are also part of the patient engagement continuum in the framework for patient engagement in health‐care policy.^8^ PPI mechanisms are characterized by degree of participant involvement, direction of information flow and representativeness of the population engaged (Figure 1). Consultation involves a unidirectional flow of information and opinions from patients and the public to a guideline development group, often through focus groups, surveys or public comment. With consultation strategies, there is typically no back‐and‐forth interaction between patient and public stakeholders and the guideline development group. In contrast, participation is achieved by including one or more patient or public stakeholders on a guideline development group alongside professional members (Figure 1). There is a bidirectional exchange of information, allowing all stakeholders to actively participate in deliberations and fostering mutual influence between stakeholder types. There is also development of a collective perspective.^6^, ^7^, ^8^

Consultation and participation strategies have distinct strengths and limitations. Consultation strategies collect a variety of perspectives from a large group of people, but fail to recognize patient and public representatives as development partners or give them an active voice in the process. The views or suggestions offered through consultation may not undergo deliberation or inform decision making. Participation strategies recognize the unique expertise of patients and the public and facilitate mutual learning and compromise. However, they rely on involvement of a small number of representatives, potentially missing the perspectives of uninvolved parties.^7^

A taxonomy for stakeholder engagement in patient‐centred outcomes research defined engagement as involving a bidirectional relationship,^9^ thus excluding consultation strategies. However, guideline developers report that recruitment difficulties,^10^ representativeness of selected participants,^10^ training and support needs,^3^, ^11^, ^12^ uncertainty of how to incorporate patient experiences,^11^, ^13^ patient representatives’ feelings of isolation,^10^ and difficulty with medical terminology and systematic review participation^1^, ^3^, ^10^, ^11^, ^12^ are all barriers to successful participation. Employing multiple engagement strategies in guideline development may allow developers to capitalize on the strengths of each approach and avoid the limitations inherent in a single strategy,^7^, ^14^ but insufficient resources are also a known barrier to PPI in guideline development^10^, ^11^ and could limit developer willingness to select certain or multiple strategies.

Given that minimal research has evaluated the relative contributions of different PPI strategies to guideline development, we aimed to assess the effect of participation and consultation on guideline question development. The goal was to identify the impact that participation and consultation strategies had on the first guideline step—question development—for a single guideline using multiple PPI approaches, in order to inform guideline developers considering incorporating PPI in their processes. Here, we report the final results of the overarching study, including previously undescribed results of public comment (consultation), determination of final guideline questions by a guideline development group including patient representatives (participation), and an assessment of the impact of participation and consultation on guideline question development.

## 2 METHODS

### 2.1 Context and previously reported findings

This study centred on question development for an American Academy of Neurology (AAN) guideline regarding the use of amyloid positron emission tomography (PET) imaging in patients with or at risk for dementia. Amyloid PET scans show the presence of amyloid plaques—one of the pathologic (autopsy) hallmarks of Alzheimer's disease (AD)—in the brains of living individuals. However, the scans cannot diagnose the clinical condition of dementia. Research shows that people desire amyloid testing results even with uncertain implications,^15^, ^16^ but consensus‐based appropriate use criteria recommend limiting scans to individuals with unexplained mild cognitive impairment (MCI), possible AD dementia with unusual features or an unusually early dementia onset.^17^ The guideline was chosen from the AAN’s waiting list of nominated and prioritized guideline projects.^18^ The US‐based AAN takes a patient/stakeholder approach to PPI rather than seeking public/consumer representatives.

**Figure 2:**
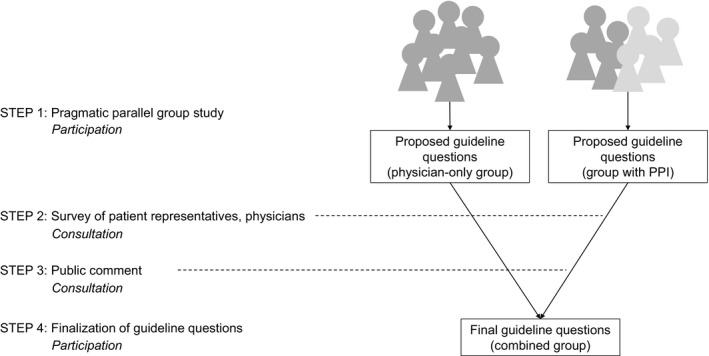
Study design and engagement strategies. Light grey: Patient and public stakeholders, dark grey: professional stakeholders. PPI: Patient and public involvement. The overarching study design included four steps, two of which involved participation PPI strategies (Steps 1 and 4) and two of which involved consultation strategies (Step 2 survey, Step 3 public comment). Further details and results of Steps 1^20^ and 2^21^ were reported previously. Two separate question development groups proposed guideline questions in Step 1; these groups combined to determine the final guideline questions in Step 4 after considering input from the preceding three steps

The overarching study design was a multiple‐methods approach (Figure 2). In Step 1, neurologists were randomly assigned to a physician‐only question development group or a question development group including physicians and patient representatives (an individual with MCI/early AD and his spouse, the spouse of an individual with dementia and a patient advocate from the Alzheimer's Association), reflecting a participation strategy. Both 8‐participant groups developed draft guideline questions using the Population‐Intervention‐Comparator‐Outcome‐Time (PICOT) framework.^19^ Proposed populations included individuals over 65 years of age, at increased risk of AD (eg due to genetics or family history), with subjective cognitive complaints, with MCI or with typical or atypical dementia. Proposed guideline questions, benefits and harms were largely similar between groups, but only the group with PPI proposed outcomes relating to progression rates and developing cognitive impairment at specific future time points (Table S1). PPI influenced meeting conduct, guideline scope, inclusion of patient‐relevant topics, outcome selection and planned approaches to recommendation development, implementation and dissemination.^20^

In Step 2 (Figure 2), investigators created a survey based on the guideline populations, outcomes and harms proposed in the PICOT questions from both question development groups, a consultation strategy. The survey was disseminated to all guideline group members, anyone providing feedback during public comment, AAN members, and patients, caregivers and dementia advocates. The survey thus reflected both PPI and professional stakeholder involvement. The patient representatives participating in question development accounted for four of the patient representative responses on the survey; two patient representatives accessed the survey from the public comment process. The majority of patient representatives for the survey were recruited through dementia, patient engagement and survey organizations.^21^ Patient representatives (n = 107) rated all items as equal to or more important than clinicians (n = 114) except one item about the potential harms of false‐positive diagnoses. Patient representatives were more concerned regarding the potential for false‐negative diagnoses across questions, whereas clinicians were more concerned regarding false‐positive diagnoses. Patient representatives also indicated that asymptomatic individuals were an extremely important population to include in the guideline, whereas physicians ranked this population as low importance.^21^

Steps 3 (public comment, a consultation strategy) and 4 (finalization of guideline questions, a participation strategy) are described further below as part of the current case study approach and analysis.

### 2.2 Current approach

This final part of the overarching study employs an instrumental case study design. Case studies are an ideal approach for performing detailed, multifaceted explorations of complex issues in real‐life contexts. Instrumental case studies (as opposed to intrinsic and collective case studies) are the approach chosen when using a particular case to gain an improved understanding of a specific issue or phenomenon.^22^, ^23^ This study uses the case of the AAN guideline on ordering amyloid PET in patients with or at risk for dementia to investigate the effect of both participation (Figure 2, Steps 1 and 4) and consultation (Figure 2, Steps 2 and 3) PPI strategies on guideline question development.

### 2.3 Step 3: public comment

Posting of guideline protocols for a 30‐day public comment period is part of standard AAN guideline methodology^18^ and reflected one of the consultation strategies for the case study. This public posting addresses the IOM standards for both external stakeholder review and public comment.^2^ The public comment posting allows for feedback from diverse stakeholders including professionals (within and outside the AAN), patient representatives and the broader public, though feedback is primarily solicited from professionals and patient representatives with relevant experience. A guideline methodologist drafted the protocol to be accessible to clinicians and members of the public. The protocol included an introduction/rationale for the guideline, proposed PICOT questions from both question development groups, proposed inclusion/exclusion criteria for studies to be included in the guideline, key definitions, funding information, the proposed author panel, author panel disclosures, and the AAN guideline conflict of interest policy and disclaimer. All members from both question development groups had the opportunity to review and approve the draft protocol. The standard AAN public comment response form asked respondents if they agreed with the proposed clinical questions and guideline approach (eg inclusion/exclusion criteria, search strategy) and provided the opportunity for free‐text comments.

The protocol was posted on the publicly available AAN website for comment on 10 March–10 April 2017 (ie there was no member firewall). The AAN announced the public comment period to AAN members via a community‐wide email and to other stakeholders by notifying the American Geriatric Society, Alzheimer Association and Alzheimer Foundation of America. The AAN invited these organizations to share information about public comment through their individual channels. At the conclusion of the public comment period, AAN staff compiled all public comment responses into a summary spreadsheet for the question development groups to review and consider when determining the final guideline questions.

### 2.4 Step 4: Drafting final guideline questions

All participants involved in the two original question development groups were invited to return for a full‐group meeting at AAN headquarters (Minneapolis, MN) on 28 July 2017. At this in‐person meeting, the PI presented the initial PICOT questions drafted by each group, a summary of the public comment responses and survey results. Panel members discussed and determined the final PICOT questions for the guideline, reflecting PPI via participation. The meeting was audio‐recorded with participants’ knowledge. Audio files were professionally transcribed verbatim.

### 2.5 Analysis

Data collected included public comment responses and the transcribed recording of the combined question development group meeting where participants selected final guideline questions. Analysis was descriptive. As the intent of this final analysis was to assess the effect of consultation and participation strategies and not to identify specific themes, a formal qualitative analysis of the retreat discussion was not performed. The PI, an investigator with an interest in guideline methodology and patient engagement, independently analysed the retreat transcript. The investigator identified retreat quotes regarding how participants incorporated the results of the participation and consultation strategies into final decisions about guideline questions. This reflected a ‘thick description’ approach,^24^ where participants’ own words were used to describe and interpret their decision making for final guideline questions. Quotes were presented verbatim and categorized according to the published conceptual model of outcomes of PPI in guideline development.^20^ This model divides the outcomes of PPI in guideline development into organizational (developer) and guideline‐specific outcomes. Organizational/developer outcomes include:

- Culture of patient‐centredness (shaping how discussions are conducted, setting patient‐centred scope, highlighting personal aspect of disease and impacting how professional team members view PPI); and
- Meaningful and effective partnerships.

Guideline‐specific outcomes include:

- Guidelines relevant to patients, stakeholders (identifying issues that may be overlooked by medical professionals, helping select patient‐relevant topics and outcomes, and influencing guideline structure/development, including language and recommendations); and
- Facilitating dissemination and implementation (education and support tools for patients and caregivers, contributing to patient guideline versions and encouraging shared decision making and active dissemination).^20^

Results were reviewed and discussed with co‐investigators. The impacts (as per the conceptual model) of PPI through the public comment (consultation) and final question development (participation) were compared to the impacts reported from earlier analyses regarding initial question development (participation) and the survey (consultation).

### 2.6 Ethics

All question development group and survey participants consented to study activities. Public comment responses were summarized as part of the standard guideline development process.

## 3 RESULTS

### 3.1 Public comment results (Step 3)

Fifty‐four individuals responded during public comment: 35 neurologists, one neuroradiologist, one research scientist and 17 individuals who responded anonymously and declined to identify their background. At least two anonymous respondents were patient or public stakeholders, as two public comment respondents participating in the survey were identified as patient stakeholders. A group of clinician‐researchers also sent a letter to the AAN during the public comment period. Because patient stakeholders did not identify themselves in the online public comment form, their responses could not be compared to those of professional respondents.

Most public comments relating to the PICOT questions focused on the proposed populations and outcomes. Multiple respondents voiced concern about the inclusion of ‘pre‐clinical’ (asymptomatic) populations without cognitive symptoms, stating that amyloid PET is not approved for this population, it is outside the scope of the appropriate use criteria,^17^ and such testing is ‘ahead of its time’. Respondents advised highlighting MCI subtypes and specifying subgroups such as individuals with known ApoE genetic status (associated with AD risk), various dementia subtypes and dementia presentations complicated by depression or comorbid conditions.

For outcomes, public comment respondents expressed concern regarding the PICOT questions looking at the prognostic (rather than diagnostic) value of amyloid PET based on neuropathologic reasons (amyloid levels plateau in early stages of AD), the perceived lack of data to answer these questions and differences in individual progression rates that may not be captured in group‐level data. Other comments covered topics such as using alternate diagnostic modalities, clarifying methods of determining a ‘positive’ scan, emphasizing the value of a negative scan for diagnostic purposes and better framing the comparator. One respondent questioned the use of amyloid PET at all given the lack of disease‐modifying therapies for AD. Another questioned the value of an evidence‐based guideline when consensus‐based guidelines already exist.

### 3.2 Results of second guideline question development meeting (Step 4)

Of 16 question development group members and two methodologists involved in the original retreat (n = 18), 12 question development group members and both methodologists attended the second question development retreat in person (n = 14). One content expert and one guideline subcommittee member attended by teleconference (Table 1, total n = 16). Review of the initial PICOT questions proposed by each group, public comment responses and survey responses occurred over 1 hour, 9 minutes. Subsequent discussion and refining of PICOT questions occurred over 4 hours, 43 minutes.

**Table 1:** Demographics of participants in second guideline meeting

Characteristic	Number (%) (n = 16)
Gender (male)	9 (56%)
Race	
White	14 (87.5%)
Other	2 (12.5%)
Age (y)	
30‐40	2 (13%)
40‐50	5 (31%)
50‐60	5 (31%)
60‐70	1 (6%)
>70	3 (19%)
Role	
Patient representative	4
Content expert	3
Guideline subcommittee member with content expertise	3
Guideline subcommittee member without content expertise (includes the two facilitators)	4
Methodologist	2

The topics covered during this meeting were different from those of the original guideline question development retreat. At this second meeting, the goal was to develop final consensus guideline questions from those originally proposed. Participants merged the initial questions and incorporated survey and public comment responses (Table S2). Much of the conversation focused on adding the correct nuances to the PICOT questions. As such, the bulk of the transcripts (not including training by and questions to the PI) reflected discussions from the two moderators (30.6%) and the content experts (three specifically invited for the guideline, 40.0%; three guideline subcommittee members with content expertise, 11.9%). The two methodologists, four patient representatives and two guideline subcommittee members without content expertise accounted for a smaller fraction of the transcripts (9.4%, 7.7% and 0.5%, respectively).

Despite the fact that the patient representatives accounted for less than 10% of the retreat transcript, there was evidence that the participation of the individual with MCI/early AD, two caregivers and the dementia advocate affected guideline development as described in the conceptual model^20^: they shaped how discussions were conducted, helped set a patient‐centred scope, highlighted the personal aspect of the disease, identified issues that might be overlooked by medical professionals and helped select patient‐relevant topics and outcomes (Table 2).

**Table 2:** Examples of ways participation affected guideline question development

PPI guideline contribution	Exemplar quote
Shaping how discussions are conducted	I have a question just I’m hearing, you know, tau and tangle, are they interchangeable or are they two different things?… You know, for the lay person, that's really nice to know… (Caregiver 1) We need to frame the guideline questions in terms of what people are actually trying to ask based on these studies. Like patients, families, clinicians. (Expert 1)
Setting patient‐centred scope	So the importance to the family is how far along is this, how prognosis will help us to plan. Just like when [name] and I both retired, we bought a home, thinking that, okay, we'll live in this home probably until our early 80s, at which time we will look into continuing care, blah, blah, blah. Now, with the MCI early‐stage Alzheimer's disease, how does that impact us, and if we did have more information, it would help in that long‐term planning process financially with our own lifestyle. So that's where the PET scan—how is that going to inform and continue to inform the patient and family? (Caregiver 1) My husband never qualified because the doctor didn't think he had anything and so, now, that he does have [an Alzheimer's disease] diagnosis, now he's in five studies, new studies. That makes a huge difference in like his sense of feeling he's making a difference as well (Caregiver 2)
Highlighting personal impact of disease	My husband's results of the PET scan were… positive… So from a not logical, from an emotional point of view, for the family, so far, we can understand him so much better, and it has made life much more pleasant, and I’m not as critical, and I’m not as on edge, and the acceptance that we have of his behaviors has made a huge, huge difference on my blood pressure as well (Caregiver 2)
Identifying issues that may be overlooked by medical professionals	I’d like to once again demonstrate the difference between memory and cognition. I don't remember if I’ve said this before but to me, this comes all down to cognition, recognizing cognition is different from memory and I don't think my practitioners practice that way. They lump it together and call it cognitive and I live it and its memory and understanding. If I don't separate those two functions, I cannot function and so lumping it together and especially to me, they call it mild cognitive impairment because that doesn't describe my problem at all. (Patient) This question came up at the Meeting of the Minds recently in terms of the general population. A woman present in one of the sessions with Mayo doctors wanted this test, and the concern was the cost. And I’ve been on [committee name], and that is, every time I talk about this, the PET scan, they said, ‘But the cost’. So that's a factor in terms of who's going to pay for this. It was also a factor for the Center for Memory and Aging as to, okay, if Medicare is going to pay for this, but who's going to pay for the time to analyze it with our doctors and so forth? So cost is definitely a factor. (Caregiver 2) I know for a fact that my husband's lifestyle has made a difference in his living with the disease, and we're now in the 23rd year of his living with the disease, and he is still functioning. He's still functioning. He's at home and he needs his caregiver at home, but his daughter has him today. But it does, lifestyle does make a huge, huge difference. (Caregiver 2)
Helping select patient‐relevant topics and outcomes	From my point of view as a person with the disease, to me, I would far rather start out on a treatment for something and find out that I didn't need it than find out later that I could have had some treatment that would have slowed a disease like this that would destroy my ability to function. So the need to know far outweighs any concerns about false positives to me (Patient) It may allay some fears from the public if we're at least trying to—showing that we're considering this issue that even if [amyloid PET] is predictive that we would [also] like to know how it actually impacts people's lives. (Expert 2) [Knowing expected prognosis] is critical for sustaining, sustenance of emotional health (Caregiver 2) Right, so, I think there's, you know, there's the additional thing, you know, that and I think we clinicians maybe undervalue this, but the value of the label for the patient may not be actionable on our part but that might have—this gets a quality of life and outcomes as well (Expert 2)

Public comment affected group discussions and nuances of the final guideline questions. The public comment suggestions to exclude asymptomatic individuals from the guideline questions prompted discussions about this topic and how the final guideline could be affected by this decision:

I think the controversy from public comments… is that ‘We should never recommend this. This is a terrible idea’. But as we’ve already said, bringing it up as a question in the guidelines doesn’t mean we’re going to recommend it. In fact, very well it’s likely we would recommend against using it in this population, which could be, I think, very helpful to practitioners. (Moderator 1)

Final decisions regarding inclusion of asymptomatic populations and prognostic questions were made after discussion of survey results (below). Public comment also resulted in discussion about how to capture the value of a negative amyloid PET scan and choosing optimal comparators:

‘What is the reason to do the test’ is to get the emphasis on the negative test in the public comment… [This relates to] this idea of your changing your clinical diagnosis. (Guideline subcommittee member/content expert 2)

All of our questions thus far are compared to no amyloid PET so, we had standard evaluation and standard evaluation plus PET… I think some of the comments who are – public comments who already defines family [practice] evaluation, that kind of thing. So, I guess we will decide… [for] which of these questions do we need to have an active comparator? (Moderator 2)

While not every public comment suggestion was followed in the selection of the final guideline questions, the question development group discussed all the comments and incorporated public comment suggestions into question wording. Comments relevant to recommendation drafting were saved for that stage of guideline development. While public comment affected conduct of the guideline question retreat, this did not end up being a successful PPI consultation strategy given the lack of patient and public respondents.

Retreat discussions demonstrated the impact of the survey (consultation) on guideline development and how professional team members view PPI. Professional participants saw survey results as confirming patient‐centred themes from the original question development meeting: the importance of a diagnosis (even if no disease‐modifying treatment is available), the importance of prognosis, including expected rate of decline, relevance of the test for asymptomatic individuals, and the potential value of knowing the amount of amyloid detected and the results of serial imaging.

The survey also indicated this [inclusion of asymptomatic individuals] is very important to the patient population, to the patients that we serve, so how can we not ask it? (Methodologist 2)

Just broadly, I would say that that finding [that patient representatives on the survey were more concerned about false negative than false positive diagnoses], I think, really emphasizes the importance of patient engagement and involvement. (Moderator 1)

I think that from what we saw in the patient and caregiver comments, both from in the group last year and also in the survey, that rates of decline are something you ought to say to the family, so I think we should decide what evidence there is for the impact of this study on predicting rate of decline. And it may be that we can’t find any or it may not be different than just accurate diagnosis, but I think we need to ask that question and see what the evidence currently available. (Expert 2)

I think it’s important that we ask the question about how predictive amyloid is, even in the late stages of progression, just to the point you’re making, and also for how important people thought it was in the public, you know, how much amyloid there is in the brain. So if we ask that question and our evidence‐based view shows that at a certain point, it’s not important to know how much amyloid is or isn’t predictive of progression compared to just diagnosis, that’s important information to have in the guideline as well for clinicians to be able to advise families and patients. (Expert 1)

With the survey results confirming the themes raised by patient representatives in the first guideline question development retreat, the question development group opted to keep guideline questions regarding the use of amyloid PET in asymptomatic populations, even though these populations are outside the appropriate use guidelines,^17^ of low value to physician survey participants^21^ and recommended against by professional public comment respondents.

PPI via participation (Figure 2, Steps 1 and 4) and consultation using a survey (Figure 2, Step 2) resulted in both similar and distinct benefits (Table 3). Given the pre‐specified questions and unidirectional nature of the survey (provision of opinions from survey respondents to the question development group), survey responses could not highlight personal experiences, actively shape in‐person discussions or build meaningful and effective partnerships. Survey responses did, however, have benefits overlapping with those offered by participation (Table 3). Additionally, the views of the large group of patient stakeholders responding to the survey confirmed the views voiced by the four question development group participants.

**Table 3:** Contributions of participation and consultation strategies in guideline question development

Contribution	Participation	Consultation
Shaping how discussions are conducted	✓	
Setting patient‐centred scope	✓	✓
Highlighting personal impact of disease	✓	
Impacting how professional team members view PPI	✓	✓
Identifying issues that may be overlooked by professionals	✓	✓
Helping select patient‐relevant topics and outcomes	✓	✓
Confirming opinions of, and relevance to, a large group of patient stakeholders		✓

### 3.3 Limitations of study PPI mechanisms

There were several limitations and barriers to PPI in this case study. In the second guideline retreat, the patient representatives raised several topics not clearly linked to the guideline. These included the patient's perceived distinction between memory and cognition and the influence of lifestyle changes on cognition and dementia. While these topics could be seen as patient stakeholders identifying issues that may be overlooked by medical professionals (Table 2), they could also be interpreted as an unnecessary use of time. Professional panellists also used a small amount of retreat time to answer the patient representatives’ technical questions. Developer time was dedicated to creating a patient‐ and public‐friendly guideline protocol to post for public comment, but the majority of public comment respondents were identified as professionals. The survey was a more successful consultation strategy but required extra resources for development and dissemination.

## 4 DISCUSSION

Conduct of the second guideline development retreat group confirmed the previously reported benefits of participation PPI strategies including shaping how discussions are conducted, setting a patient‐centred scope, highlighting the personal aspects of disease, affecting how professional team members view PPI, identifying issues that might be overlooked by medical professionals and selecting patient‐relevant guideline topics and outcomes.^20^ Public comment as a consultation strategy affected discussions and final question decisions, but reflected input from professionals more than the public or patient representatives. Use of a survey as a consultation strategy, however, confirmed the priorities voiced by the patient representatives on the guideline panel. Survey responses emphasized the importance of having a dementia diagnosis, including asymptomatic populations in the guideline and looking at progression as a guideline outcome.

The relative impact of patient representative versus professional/clinician members on final question development is difficult to assess given multiple combined approaches to PPI, different expertise brought by question development group members to the retreat discussion, and reliance on meeting transcripts. The retreat moderators, clinicians with content expertise and guideline methodologists accounted for 91.9% of the transcript discussion, so they clearly had a large role in influencing discussions and in providing expert nuance to the final selected PICOT questions. However, transcript quotes (Table 2, text) clearly demonstrate that professional question development group members were influenced by the views of the patient representatives in the room and the survey respondents.

Participation and survey consultation approaches to PPI resulted in similar but distinct benefits (Table 3). The survey responses were particularly helpful for reinforcing that a large group of patient stakeholders agreed with the views voiced by the four question development group participants. This helped mitigate the limitation associated with participation strategies—the fact that a small group of participants may not represent the broader population. This consultation benefit was particularly important given that the patient representative views were contrary to the professional opinions provided during the public comment period. Multiple professional public commenters recommended excluding asymptomatic individuals from the guideline and expressed concern regarding the PICOT questions looking at the prognostic value of amyloid PET. Given that guideline developers are often looking for ways to reduce guideline scope to help limit the time and resources needed for successful guideline completion, it is plausible that these public comments could have resulted in removal of guideline PICOT questions if not for the multiple PPI approaches emphasizing consistent patient stakeholder views.

The complementary benefits of participation and consultation strategies for question development in this case study—and the increased weight from consistent views across multiple strategies—support the use of multiple approaches for optimal PPI.^7^, ^14^ This is consistent with IOM standards recommending inclusion of a current or former patient and a patient advocate or patient/consumer organization representative on guideline development panels, external review including patients and representatives of the public, and public comment.^2^ We obtained more meaningful patient stakeholder feedback from a survey than from public comment, however, with only two anonymous members of the public responding to the public posting. Guideline developers desiring broad representation for meaningful consultation will likely need to choose active, targeted approaches (eg survey, focus groups) rather than passive ones (public comment posting). Alternatively, developers may need to identify improved strategies for disseminating information regarding public comment opportunities or incentives for patient representative participation.

This study employed multiple strategies to overcome known barriers to successful PPI, which may affect study generalizability. Investigators used the patient and service user engagement in research framework^25^ and pilot focus groups^26^ to develop initiation strategies (eg early involvement, engaging interested populations, pre‐training), promote co‐learning (eg through shared education at both question development retreats, use of skilled moderators), and build reciprocal relationships (eg through engaging multiple stakeholders, treating patient representatives as equals and acknowledging the importance of different roles). To overcome recruitment difficulties^10^ and limit the resources needed for successful engagement,^11^ investigators asked the local chapter of the Alzheimer's Association to identify interested stakeholders who could attend the two in‐person meetings. Patient stakeholders thus had at least a basic knowledge of the relevant medical terminology, and professional participants were sensitive to explaining technical considerations and answering questions, thus addressing the barrier of medical jargon.^1^, ^3^, ^10^, ^12^ To address the concern of medical jargon affecting consultation strategies, a plain‐language version of the guideline protocol was posted for public comment and AAN survey staff reviewed the survey for readability.

Strengths of the case study include a systematic assessment of the effect of participation and consultation strategies during the course of guideline question development and the use of the previously developed conceptual framework.^20^ Study limitations include the fact that the case was confined to the first step of guideline development, selecting the guideline questions. Additionally, participants knew that they were in a study investigating the effects of PPI on guideline development, so they may have been more inclusive of patient and public views than in other circumstances. The study reflects experience with a single guideline, and several strategies were utilized to optimize PPI, so results will not be fully generalizable. The use of multiple PPI strategies also precluded assessment of the relative benefits and limitations of these strategies in isolation. Reliance on retreat transcripts required interpretation of the influence of PPI from participant quotes. Because it combined participants from the two earlier parallel groups, the second retreat group size was larger than desirable. Despite this, patient representatives were comfortable enough to make meaningful contributions to question development. The stakeholders recommended by the Alzheimer's Association were highly educated and engaged; their experiences and contributions may not generalize to other populations. Data were acquired only during the step of question development, limiting conclusions regarding the impact of PPI at other guideline steps. Finally, relevant costs were shared between the AAN and research funds and thus do not represent regularly available resources, also potentially limiting generalizability. AAN staff routinely edit guideline protocols for public comment posting and summarize public comment results, and the AAN owns survey software. The supporting grant and additional research funds were used to pay for the two question development retreats (including participant travel), staff time for study‐related activities including engaging with the panel, organizing the in‐person retreats, and survey development and implementation, and investigator responsibilities and analyses.

## 5 CONCLUSIONS

This study showed that while public comment was not successful in obtaining views of a large representative group of patient and public stakeholders, use of a survey as a consultation PPI strategy and active patient stakeholder participation on a question development group both had important effects on guideline question development. Whereas participation gave patient representatives an active voice on the development group and fostered bidirectional exchanges with professional members, survey responses confirmed the views of the question development group with a broader population. The combined approach resulted in patient‐centred guideline questions including populations and outcomes promoted by patient representatives despite negative feedback from professional public commenters. Consultation and participation PPI strategies are not interchangeable. Guideline developers should prioritize using both participation and active consultation strategies to successfully engage patient representatives and public stakeholders.

## CONFLICT OF INTEREST

MJ Armstrong is supported by an ARHQ K08 career development award (K08HS24159) through which the current study was performed. She also receives research support from a 1Florida ADRC (AG047266) pilot grant, as the local PI of a Lewy Body Dementia Association Research Center of Excellence, and from the Michael J. Fox Foundation. She receives compensation from the AAN for work as an evidence‐based medicine methodology consultant. G. S. Gronseth receives compensation from the American Academy of Neurology for work as an evidence‐based medicine methodology consultant, as an editorial board member for the patient magazine *Brain & Life* and for work as an associate editor of *Neurology*. C. D. Mullins receives grant support from Merck and consulting income from Bayer, Boehringer Ingelheim, Illumina, Pfizer, Sanofi and Regeneron. A. R. Gagliardi declares no conflicts of interest.

## ETHICAL APPROVAL

The University of Florida Institutional Review Board provided approval for the study (IRB201501210).

## Data Availability

The qualitative data (de‐identified retreat transcripts) that support the findings of this study are available from the corresponding author upon reasonable request. Participant identities are discoverable, and thus, the transcripts are not publicly posted. Guideline‐related information (eg the summary of public comment responses) is available through the American Academy of Neurology upon reasonable request.
