# Supplementary material for: Participation and consultation engagement strategies have complementary roles: A case study of patient and public involvement in clinical practice guideline development
Source: Health Expect. 2019 Dec 29;23(2):423–32. doi: 10.1111/hex.13018 (PMC7104634; doi:10.1111/hex.13018)
Supplement: Supplementary file 1 [file HEX-23-423-s001.docx]

**Table e-1. PICO Questions from First Retreat**

Gp 1: Experimental group (including patient representatives) – black text; Gp2: Control group (consisting of all physicians) – blue text

|  | Gp 1 Q1 | Gp 1 Q2 | Gp 1 Q3 | Gp 1 Q4 |
| --- | --- | --- | --- | --- |
| P | In patients at risk for AD without CI (preclinical), | In patients with MCI suspected to be prodromal AD, | In patients with dementia, | In patients with dementia, |
| I | Does amyloid imaging | Does amyloid imaging | Does amyloid imaging | Does amyloid imaging |
| C | Compared to not getting beta-amyloid imaging | Compared to not getting beta-amyloid imaging | Compared to not getting beta-amyloid imaging | Compared to not getting beta-amyloid imaging |
| O | Accurately predict developing cognitive impairment due to Alzheimer’s pathology | Accurately predict developing dementia due to Alzheimer’s pathology | Improve accuracy of diagnosing Alzheimer’s disease dementia? | Accurately predict rate of decline due to Alzheimer’s dementia? |
| T | In 1, 3, 5, 10, and 15 years? | In 1, 3, 5, 10, and 15 years? |  |  |

|  | Gp 1 Q5 | Gp 1 Q6 | G2 Q2 | G2 Q3 |
| --- | --- | --- | --- | --- |
| P | In patients with MCI, | In patients with dementia, | For persons at present or future risk of ADD (1. asymptomatic, 2. subjective symptoms without objective findings, 3. MCI, 4. typical dementia [AD], 5. atypical dementia [AD], | For persons at present or future risk of ADD (1. asymptomatic, 2. subjective symptoms without objective findings, 3. MCI, 4. typical dementia [AD], 5. atypical dementia [AD], |
| I | Does beta-amyloid imaging | Does beta-amyloid imaging | Does amyloid PET | Does amyloid PET in addition to “standard evaluation” (standard clinical, radiographic, CSF evaluation) |
| C | Compared to other testing (see list; head-to-head comparisons only) | Compared to other testing (see list; head-to-head comparisons only) | As compared to a reference standard (see list) | As compared to a standard evaluation alone |
| O | Better predict progression to Alzheimer’s dementia? | Better determine the type of dementia? | Accurately identify patients with ADD as determined by an independent reference standard (the outcome here will be sensitivities, specificities and for non-case control studies predictive values) | Increase the accuracy of identifying patients with ADD as determined by an independent reference standard (outcome: comparison of diagnostic accuracy measures) |
| Com-ment |  |  | Direct harms of testing will also be sought | The added element is to directly compare the accuracy of diagnosis with and without amyloid PET |

|  | Gp 1 Q7 | Gp 1 Q8 | Gp 2 Q1 | Gp 2 Q4 |
| --- | --- | --- | --- | --- |
| P | In patients with dementia, | In patients at risk for AD without CI (preclinical), | For persons at present or future risk of ADD (1. asymptomatic, 2. subjective symptoms without objective findings, 3. MCI, 4. typical dementia [AD], 5. atypical dementia [AD], | For persons without ADD |
| I | Does beta-amyloid imaging | Does beta-amyloid imaging | Does undergoing amyloid PET (any tracer) | How often does amyloid PET |
| C | Compared to other testing (see list; head-to-head comparisons only) | As compared to other testing (see list; head-to-head comparisons only) | Compared to not undergoing amyloid PET | (As compared to how often it would be indentified without amyloid PET, which is zero by definition) |
| O | Better predict rate of progression? | Better predict progression to Alzheimer’s dementia? | Improve outcomes:  - Quality of life  - Overall costs  - Other outcomes (benefits/harms of “correct” diagnosis, stopping search for other causes, treatment, preparation for personal consequences of ADD, preparation for social consequences of ADD) | Demonstrate amyloid? (Proportion of persons with amyloid) |

AD: Alzheimer disease, ADD: Alzheimer disease dementia, CI: cognitive impairment, MCI: mild cognitive impairment, PET: positron emission tomography

**Table e-2. Final PICOT Questions (After Retreat #2)**

| **Ques. #** | PICO Question |
| --- | --- |
| Question 1: Test characteristics | |
| 1a | P: In persons who undergo amyloid PET and are described as having a “positive” scan  I: (using different tracers)  C: compared to those described as having a “negative” scan  O: what is the frequency of neuropathologic-confirmed amyloid? |
| 1b | P: In persons undergoing amyloid PET  I: how often do patients with “positive” scans  C: versus those with “negative” scans  O: have Alzheimer’s disease as determined by a clinicopathologic standard?  Comment: Alzheimer’s disease is not diagnosed based on amyloid alone, so the outcome here is not simply dependent on the presence of amyloid. Also, some patients will have pathology consistent with Alzheimer’s disease but not a clinical syndrome. |
| Question 2: Population of people without signs or symptoms of cognitive impairment | |
| 2a-i | P: In people without signs or symptoms of cognitive impairment  I: does a positive amyloid PET  C: compared to a negative amyloid PET  O: identify people at increased risk for developing cognitive impairment (MCI, dementia [ADD or other dementia])  T: at specific times post imaging?  Comments:  - evidence will guide time frame  - relevant outcomes may include MCI, dementia, or cognitive test scores (etc) |
| 2a-ii | P: For clinicians evaluating people without signs or symptoms of cognitive impairment  I: does knowledge of the results of an amyloid PET  C: compared to no knowledge of the results of the amyloid PET  O: improve the clinician’s accuracy in predicting which patients will develop ADD |
| 2b-i | P: In people without signs or symptoms of cognitive impairment  I: does undergoing amyloid PET  C: compared to not undergoing amyloid PET  O: improve or worsen outcomes such as quality of life, patient distress, caregiver distress, anxiety, depression etc. |
| 2b-ii | P: In people without signs or symptoms of cognitive impairment  I: does knowing amyloid PET results  C: compared to not knowing amyloid PET results  O: improve or worsen outcomes such as quality of life, patient distress, caregiver distress, depression, anxiety, etc. |
| 2b-iii | P: In people without signs or symptoms of cognitive impairment  I: does having a positive amyloid PET  C: compared to having a negative amyloid PET  O: improve or worsen outcomes such as quality of life, patient distress, caregiver distress, depression, anxiety, etc. |
| 2c | P: In people without signs or symptoms of cognitive impairment  I: does a worsening trajectory on *serial* amyloid PET scans  C: compared to a stable trajectory  O: improve prognostic accuracy for the development of cognitive impairment (MCI, dementia [ADD or other dementia])  T: at specific times post imaging?  Comment: evidence will guide time frame |
| Question 3: Population of subjective cognitive complaints (SCC) | |
| 3a | P: In people with SCC and a normal cognitive exam (as defined in the studies included)  I: does a positive amyloid PET  C: compared to a negative amyloid PET  O: identify people at increased risk for developing cognitive impairment (MCI, dementia [ADD or other dementia])  T: at specific times post imaging?  Comments:  - evidence will guide time frame  - relevant outcomes may include MCI, dementia, or cognitive test scores (etc) |
| 3b-i | P: In people with SCC and a normal cognitive exam (as defined in the studies included)  I: does undergoing amyloid PET  C: compared to not undergoing amyloid PET  O: improve or worsen outcomes such as quality of life, patient distress, caregiver distress, depression, anxiety, etc? |
| 3b-ii | P: In people with SCC and a normal cognitive exam (as defined in the studies included)  I: does knowing amyloid PET results  C: compared to not knowing amyloid PET results  O: improve or worsen outcomes such as quality of life, patient distress, caregiver distress, depression, anxiety, etc? |
| 3b-iii | P: In people with SCC and a normal cognitive exam (as defined in the studies included)  I: does having a positive amyloid PET  C: compared to having a negative amyloid PET  O: improve or worsen outcomes such as quality of life, patient distress, caregiver distress, depression, anxiety, etc? |
| Question 4: Population of persons with MCI | |
| 4a-i | P: In people with MCI  I: does a positive amyloid PET  C: compared to a negative amyloid PET  O: identify people at increased risk for developing cognitive impairment (MCI, dementia [ADD or other dementia])  T: at specific times post imaging?  Comments:  - evidence will guide time frame  - relevant outcomes may include dementia or cognitive test scores (etc) |
| 4a-ii | P: For clinicians evaluating people with MCI  I: does knowledge of the results of an amyloid PET  C: compared to no knowledge of the results of the amyloid PET  O: improve the clinician’s accuracy in predicting which patients will develop ADD |
| 4b-i | P: In people with MCI  I: does undergoing amyloid PET  C: compared to not undergoing amyloid PET  O: improve or worsen outcomes such as quality of life, patient distress, caregiver distress, depression, anxiety, etc? |
| 4b-ii | P: In people with MCI  I: does knowing amyloid PET results  C: compared to not knowing amyloid PET results  O: improve or worsen outcomes such as quality of life, patient distress, caregiver distress, depression, anxiety, etc? |
| 4b-iii | P: In people with MCI  I: does having a positive amyloid PET  C: compared to having a negative amyloid PET  O: improve or worsen outcomes such as quality of life, patient distress, caregiver distress, depression, anxiety, etc? |
| 4c | P: In people with MCI  I: does a worsening trajectory on *serial* amyloid PET scans  C: compared to a stable trajectory  O: improve prognostic accuracy for the development of cognitive impairment (MCI, dementia [ADD or other dementia])  T: at specific times post imaging?  Comment: evidence will guide time frame |
| Question 5: Population of people with dementia | |
| 5a | P: In people with dementia (typical AD pattern, atypical AD pattern, unknown dementia type, young onset)  I: undergoing amyloid PET  O: what is the frequency of amyloid positivity/amyloid negativity? |
| 5b-i | P: In people with dementia (typical AD pattern, atypical AD pattern, unknown dementia type, young onset)  I: does a positive amyloid PET  C: compared to a negative amyloid PET  O: improve the clinician’s accuracy in predicting which patients have ADD? |
| 5b-ii | P: For clinicians evaluating people with dementia (typical AD pattern, atypical AD pattern, unknown dementia type, young onset)  I: does knowledge of the results of an amyloid PET  C: compared to no knowledge of the results of the amyloid PET  O: improve the clinician’s accuracy in predicting which patients have ADD |
| 5c | P: In people with dementia (typical AD pattern, atypical AD pattern, unknown dementia type, young onset)  I: does a positive amyloid PET  C: compared to a negative amyloid PET  O: accurately predict rate of decline?  Comments:  - evidence will guide time frame |
| 5d-i | P: In people with dementia (typical AD pattern, atypical AD pattern, unknown dementia type, young onset)  I: does undergoing amyloid PET  C: compared to not undergoing amyloid PET  O: improve or worsen outcomes such as quality of life, patient distress, caregiver distress, depression, anxiety, etc? |
| 5d-ii | P: In people with dementia (typical AD pattern, atypical AD pattern, unknown dementia type, young onset)  I: does knowing amyloid PET results  C: compared to not knowing amyloid PET results  O: improve or worsen outcomes such as quality of life, patient distress, caregiver distress, depression, anxiety, etc? |
| 5d-iii | P: In people with dementia (typical AD pattern, atypical AD pattern, unknown dementia type, young onset)  I: does having a positive amyloid PET  C: compared to having a negative amyloid PET  O: improve or worsen outcomes such as quality of life, patient distress, caregiver distress, depression, anxiety, etc? |
| 5e | In people with dementia (typical AD pattern, atypical AD pattern, unknown dementia type, young onset)  I: does undergoing *serial* amyloid PET  C: compared to not undergoing serial amyloid PET  O: accurately predict subsequent rate of decline? |

AD: Alzheimer disease, ADD: Alzheimer disease dementia, MCI: mild cognitive impairment, PET: positron emission tomography, SCC: subjective cognitive complaints/concerns
